# Occurrence of Disinfection By-Products in Swimming Pools in the Area of Thessaloniki, Northern Greece. Assessment of Multi-Pathway Exposure and Risk

**DOI:** 10.3390/molecules26247639

**Published:** 2021-12-16

**Authors:** Akrivi Sdougkou, Kyriaki Kapsalaki, Argyri Kozari, Ioanna Pantelaki, Dimitra Voutsa

**Affiliations:** Environmental Pollution Control Laboratory, School of Chemistry, Aristotle University, 541 24 Thessaloniki, Greece; akrivisd@chem.auth.gr (A.S.); kapsalaki@chem.auth.gr (K.K.); akozaria@chem.auth.gr (A.K.); ipantelaki@chem.auth.gr (I.P.)

**Keywords:** DBPs, haloacetic acids, halonitriles, trihalomethanes, swimming pool, carcinogenic risk, cytotoxicity, dermal, inhalation, ingestion

## Abstract

This study investigated the occurrence of disinfection by-products (DBPs) (trihalomethanes (THMs), haloacetic acids (HAAs), halonitriles (HANs), halonitromethane (TCNM) and haloketones (HKs)) in different type of swimming pools in the area of Thessaloniki, northern Greece by employing the EPA methods 551.1 and 552.3. Moreover, general water quality parameters (pH, residual chlorine, dissolved organic carbon, UV_254_ absorption, total nitrogen, alkalinity and conductivity) were also measured. The concentrations of DBPs showed great variability among swimming pools as well as within the same pool between sampling campaigns. HAAs exhibited the highest concentrations followed by THMs, HANs, TCNM and HKs. Exposure doses for four age groups (3–<6 y, 6–<11 y, 11–<16 y and adults) were calculated. Route-specific exposures varied among DBPs groups. Inhalation was the dominant exposure route to THMs and TCNM (up to 92–95%). Ingestion and dermal absorption were the main exposure routes to HAAs (40–82% and 18–59%, respectively), depending on the age of swimmers. HANs contributed up to 75% to the calculated cytotoxicity of pool water. Hazard indices for different exposure routes were <1, suggesting non-carcinogenic risk. Inhalation posed the higher carcinogenic risk for THMs, whereas risk via oral and dermal routes was low. Ingestion and dermal contact posed the higher risk for HAAs. Risk management strategies that minimise DBPs exposure without compromising disinfection efficiency in swimming pools are necessary.

## 1. Introduction

Disinfection is a necessary water treatment process used in swimming pools to inactivate pathogens and prevent outbreaks of infectious diseases. Chlorine is the most common disinfectant for this purpose. However, this practice also results in the formation of undesirable disinfection by-products (DBPs) from the reaction of chlorine with the organic matter present in filling waters (natural organic matter) and released from swimmers (human body fluids, sweat, sebum, skin particles, personal care products etc). Differences in operation conditions and disinfection methods affect the levels and speciation of the DBPs in swimming pools [1,2,3]. 

A large number of DBPs are considered as cytotoxic, neurotoxic, mutagenic, genotoxic, carcinogenic and teratogenic [4,5,6]. Concerns have been raised regarding potential negative effects on human health from water disinfectants used in swimming pools. The exposure to DBPs through different intake routes (inhalation, dermal absorption, water ingestion) may pose health risk for swimmers and pool staff. Links between exposure to DBPs and several health issues have been investigated [7,8,9]. Overall, the available knowledge suggests that the health benefits of swimming outweigh the potential health risks and, positive effects of swimming should be enhanced by minimizing potential risks. Although many studies reported the occurrence of commonly investigated DBPs (mainly trihalomethanes and haloacetic acids) in swimming pools, there are only a few studies that investigated emerging DBP classes, such as nitrogenous DBPs (halonitromethanes, haloacetonitriles etc.).

The aim of this study was to investigate for the first time the presence of various priority and emerging DBP groups (trihalomethanes, haloacetic acids, halonitriles, halonitromethanes and haloketones) under swimming pool operation conditions as practiced in the area of Thessaloniki, Northern Greece. DBPs as well as other water quality parameters (pH, residual chlorine, dissolved organic carbon, UV absorption at 254 nm, alkalinity, conductivity, total nitrogen) were determined during the period July 2019–February 2020. Specifically, the main objectives of this article are to:

(a) Examine the occurrence of different DBPs groups in swimming pools;
(b) Find out possible correlations among DBPs and physicochemical water parameters;
(c) Estimate the contribution of different exposure routes to DBPs and;
(d) Present a multi-pathway risk assessment for four age-groups of swimmers.

## 2. Experimental

### 2.1. Analytical Standards and Reagents

Target DBPs classes and individual species are shown in Table 1. Analytical standards for the studied DBPs were used for the purpose of this study. The analytical standard for THMs EPA 501/601 trihalomenthanes calibration mix certified reference material, 100 μg/mL each component in methanol, was obtained from Sigma-Aldrich and is a mixture of four compounds (TCM, DCBM, CDBM and TBM). Analytical standard EPA 551B Halogenated Volatiles Mix certified reference material (2000 μg/mL each compnentin methyl tert-butyl ether) purchased from Sigma–Aldrich contains four haloacetonitriles (DCAN, TCAN, BCAN, DBAN), one halonitromethane (TCNM) and two haloketones (DCP, TCP). The analytical standard EPA 552.2 Haloacetic Acids Mix certified reference material 2000 μg/mL each component in methyl tert-butyl ether was obtained from Supelco and is a mixture of nine haloacetic acids (BCAA, BDCAA, CDBAA, DBAA, DCAA, MBAA, MCAA, TBAA, TCAA). The internal standard 1,2-dibromopropane was obtained from Chem. Service (West Chester, U.S.A). The surrogate standard 2,3 dibromoproprionic acid was provided from Supelco. Methyl tert-butyl ester (MtBE), Na_2_HPO_4_, KH_2_PO_4_, Na_2_SO_4_, NH_4_Cl and methanol were provided from Sigma-Aldrich. Milli-Q water provided by the Simplicity UV Ultrapure Water System (Millipore, Molsheim, France). 

### 2.2. Water Samples

A range of swimming pool types (indoor, outdoor, only for children and for children/adults) located in the area of Thessaloniki, Northern Greece were included in this study. The type of pools and the disinfectant used are described in Table 2. In order to ensure confidentiality, samples have been identified by applying codes (SP1 to SP14). 

Water samples were collected from 14 swimming pools, consisting of 7 outdoor and 7 indoor pools, 4 for children (≤7 years) and the rest for adults/children >7 year during the period July 2019 to February 2020 (5 sampling were conducted). Unfortunately, it was not possible to have access to detailed information (e.g., number of swimmers, average daily attendance rate, frequency of pool water replacement, regularity of filter backwashing and occurrence of shock chlorination procedures). 

Sampling was conducted in evening, between 18.00 and 20.00 p.m. Equal volumes of water samples collected at the four corners of pool, from approximately 50 cm from the side walls and 30 cm below the water’s surface, were combined in order to obtain a composite sample. Prior to sampling different reagents were added to the sample vials in order to quench any chlorine residual or standardise pH values. At each pool, three bottles were filled: (1) one 60 mL headspace free amber glass bottle for determination of THMs-HANs-HNM-HKs. This vial contained NH_4_Cl as dechlorination agent and KH_2_PO_4_/NaHPO_4_ as buffer to lower pH 4.8–5.5 in order to inhibit base catalysed degradation of HANs and standardise the pH in all samples, (2) one 60 mL headspace free amber glass bottle contained NH_4_Cl as dechlorination agent for determination of HAAs and, (3) one 500 mL glass bottle for determination of water quality parameters. Water samples were immediately placed in a cooler, transported to the laboratory within 2 h and then stored at 4 °C until analysis (within 1–4 days). Tap water from the main drinking water supply system of the city was used as filling water for swimming pools. Tap water samples were also collected at selected facilities to assess their impact on the formation of DBPs in the pools. 

All glassware used for sampling and analysis was meticulously cleaned with detergent and tap water, rinsed with tap water followed by Milli-Q water and solvents.

### 2.3. Analytical Methods 

DBPs were recovered from water samples by employing various extraction methods. THMs, HANs, TCNM and HKs were extracted with MtBE according to the EPA 551.1 method [10]. HAAs were analysed according to the EPA 552.3 [11] that includes liquid–liquid extraction with MtBE followed by derivatisation (methylation) via acidic methanol. GC/ECD (Trace GC Ultra, Thermo Scientific) with an analytical column AT-5 (30 m, id 0.25 mm, 0.25 μm, Grace) was used for determination of DBPs. THM, HANs, TCNM, HKs were analysed in the same run and the GC temperature program was: 35 °C for 9 min, 1 °C/min to 40 °C (3 min), 6 °C/min to 120 °C (5 min) and then 10 °C/min to 220 (5 min). For HAAs, the GC temperature program was: 37 °C for 21min, 5 °C/min to 136 °C (3 min), and then 20 °C/min to 240 (5 min). The injector and detector temperatures were set at 210 °C and 280 °C, respectively.

Procedural standard calibration curves were used to quantify DBPs. Internal standards as per individual method requirements were employed and quantification for each analyte was based on relative response ratios. The recovery of DBPs ranged from 80–112%, precision was better than 11% and detection limits ranged from 0.14–0.50 μg/L for THMs, 0.15–0.34 μg/L for NDBPs and HKs and 0.04–0.15 μg/L for HAAs (Table S1). 

Free chlorine was measured on site upon the addition of N,N-diethylp-phenylenediamine (DPD) reagent using a portable Pocket Colorimeter (HAΝΝA instruments (HI 96, 710 and free chlorine reagent Hi37101-01). pH and conductivity were also measured on site using a portable multi-meter (Dr Lange, ECM). DOC (as nonpurgeable organic carbon) was measured and filtrated through 0.45-μm samples, using a TOC-Vcsh analyser (Shimadzu). UV absorbance at 254 nm was also measured in filtrated samples by a spectrophotometer (Hitachi U-2001). SUVA was calculated as the ratio of UV_254_ to DOC. Total nitrogen (TN) was measured after digestion of unfiltered sample by persulfate method and alkalinity by titration with sulfate acid according to standard methods [12].

### 2.4. Risk Assessment

#### 2.4.1. Assessment of Cytotoxicity 

The chronic cytotoxicity of pool waters due to the presence of the studied DBPs was evaluated based on effective concentrations of EC_50_ values (a measure of the minimum concentration of a particular compound that induces a 50% reduction in density of Chinese hamster ovary cells after 72 h) [13]. The molar concentration of each DBP was divided by its EC_50_ value for those DBP species that were available, resulting in a dimensionless cytotoxicity value. The sum of these values represent the DBP-derived cytotoxic nature of pool water. 

#### 2.4.2. Exposure Doses

Swimmers are exposed to DBPs through various routes such as incidental oral exposure, dermal exposure, inhalation buccal, aural and nasal orbital exposures according to Swimmer Exposure Assessment Model (SWIMODEL) [14]. 

The average daily dose (ADD, mg/kg-event) though ingestion (ADD_Ing_), dermal absorption(ADD_Abs_) and inhalation (ADD_Inh_) was estimated according to the following equations [15]:

Average Daily Dose through ingestion (ADD_ing_, mg/kg-day):

ADD_Ing_ = (Cw × IngR × EF × ED)/(BW × AT)

Average Daily Dose absorbed through dermal (ADD_Abs_, mg/kg-day):

ADD_Abs_ = (Cw × Kp × t × SA × EF × ED)/(BW × AT)

Average Daily Intake through inhalation (ADD_Inh_, mg/kg-day):

ADD_Inh_ = (Ca × InhR × EF × ED)/(BW × AT)

where: Cw: concentration of DBPs in water (mg/L), Ca: Concentration of DBPs in air (mg/m^3^), IingR: Ingestion rate (L/day), EF: Exposure frequency (days/year), ED: Exposure duration (years), BW: Body weight (kg), AT: Averaging time (days), Kp: Permeability coefficient (cm/h), t: Time of contact (h/event, days), SA: Skin surface area available for contact (cm^2^), InhR: Inhalation rate (m^3^/h).

When both water and air concentrations of DBPs are measured in swimming pool facilities the actual exposure doses are obtained. However, in case that air concentrations are not determined, they can be calculated from the measured water concentrations through different approaches i.e., using Henry’s Law or Raoult’s Law as in SWIMODEL or through fugacity model [14,16,17]. Dyke et al. [16] who compared measured air concentrations of THMs with those calculated by different methods reported that the Henry’s Law approach appeared to overestimate two-three orders of magnitude the experimental data. Lourencetti et al. [17] also found that the actual THMs concentrations measured in air were significantly lower than those expected from the Henry’s Law and the ratios of measured to calculated concentrations of THMs ranged from 0.6 to 5.6% for individual species (0.9–1.4% for chloroform). In our study the Henry’s Law based on equilibrium with water was used to calculate air concentrations. This approach overestimates the actual concentrations occurred in a natatorium where ventilation is continuously driving the system away from its equilibrium [16,17]. In addition, in order to estimate exposure through inhalation and possible risks, we made the assumption that actual THMs air concentrations are 2% of the calculated values, based on the findings of Lourencetti et al. [17]. The efficiency of the absorption through exposure routes was assumed to be 100%. The exposure factors employed in this study and the properties of the studied DBPs are shown in Table 3 and Table 4.

**Table 3:** Exposure factors for non-competitive swimmers [18].

Abr	Exposure Factors	Age Groups of Non-Competitive Swimmers			
		Children (3–<6 y)	Children (6–<11 y)	Children (11–<16 y)	Adults (>18 y)
BW	Body weight (kg)	19	32	57	80
SA	Surface area (m^2^)	0.76	1.08	1.59	1.94
InhR	Inhalation rate (m^3^/h)	0.66	0.66	0.78	0.74
IngR	Ingestion rate (L/h)	0.049	0.049	0.049	0.025
EF	Exposure frequency (min/month)	137	151	139	181
ED	Exposure duration (years)	4	5	5	30
AT	Average Time	78	78	78	78

**Table 4:** Properties and risk factors used for assessing exposure and health risk.

DBPs	H (atm m^3^/mol) [19,20]	Kp (cm/h) [19,20]	EC_50_ (M) [13]	Rfd (mg/kg-day) [21]	IARC [22]	SF (mg/kg-day) [21,23]		
						Oral	Dermal *	Inhalation *
**THMs**								
TCM	3.67 × 10^−3^	6.83 × 10^−3^	9.62 × 10^−3^	1 × 10^−2^	2B	1 × 10^−2^	1 × 10^−2^	1.9 × 10^−2^
BDCM	2.12 × 10^−3^	4.02 × 10^−3^	1.15 × 10^−2^	2 × 10^−2^	2B	6.2 × 10^−2^	6.2 × 10^−2^	1.3 × 10^−1^
CDBM	7.83 × 10^−4^	2.89 × 10^−3^	5.36 × 10^−3^	2 × 10^−2^	3	8.4 × 10^−2^	8.4 × 10^−2^	8.4 × 10^−2^
TBM	5.35 × 10^−4^	2.35 × 10^−3^	3.96 × 10^−3^	2 × 10^−2^	3	7.9 × 10^−2^	7.9 × 10^−2^	3.9 × 10^−3^ **
**HAAS**								
DCAA	8.38 × 10^−9^	1.21 × 10^−3^	7.3 × 10^−3^	4 × 10^−3^	2B	5 × 10^−2^	5 × 10^−2^	5 × 10^−2^
TCAA	1.35 × 10^−8^	1.45 × 10^−3^	2.4 × 10^−3^	2 × 10^−3^	2B	7 × 10^−2^	5 × 10^−2^	5 × 10^−2^
NDBPs ***								
TCAN	1.34 × 10^−6^	7.6 × 10^−3^	1.601× 0^−4^		3			
DCAN	3.79 × 10^−6^	6.5 × 10^−4^	5.73 × 10^−5^		3			
BCAN	1.24 × 10^−6^	4.1 × 10^−4^	8.46 × 10^−6^		3			
DBAN	4.06 × 10^−7^	2.5 × 10^−4^	2.85 × 10^−6^		2B			
TCNM	2.05 × 10^−3^	5.8 × 10^−3^	5.36 × 10^−4^					
HKs ***								
DCA	6.15 × 10^−6^	4.4 × 10^−4^						
TCA	2.17 × 10^−6^	1.2 × 10^−3^						

* Oral SFs were used for dermal exposure and inhalation when data were not available. ** SF was derived from inhalation unit risk. *** Kp was calculated according to the equation logKp = −2.72 + 0.71logK_ow_ − 0.006161MW.

#### 2.4.3. Non-Carcinogenic Risk 

The non-carcinogenic risk for an individual i DBP specie was assessed as hazard quotient (HQ) according to the equation HQ_i_ = $\frac{\mathrm{ADDi}}{\mathrm{RfDi}}$ where ADD_i_ (mg/kg-day) is average daily intake considering AT = ED and RƒD_i_ is the reference daily dose for the i DBP specie. RƒDs values for oral exposure are shown in Table 4. The same values were also used for assessing hazard indices through dermal and inhalation routes. Hazard Index (HI) represents the sum of hazard quotients for all DBPs.

#### 2.4.4. Carcinogenic Risk 

Lifetime cancer risk (CR_i_) for individual i DBP specie for a specific exposure route was calculated according to the equation: CR_i_ = LADD_i_ × SF_i_, where LADD_i_: is the Lifetime Average daily dose (mg/kg-day) calculated from ADD, considering AT as lifetime (in days), and SF_i_ is the cancer slope factor for individual i DBP specie. SF_i_ values are shown in Table 4. Cancer slope factors through oral exposure were used also for the other routes that SFs are not available.

### 2.5. Statistical Evaluation 

Statistical analysis was performed using SPSS Statistics version 24 software (IBM, Armonk, NY, USA). Non detectable concentrations were treated as half of LOD. Correlation among DBPs and water quality parameters was examined through non-parametric Spearman’s correlation analysis, since data are not follow normal distribution. 

## 3. Results and Discussion

### 3.1. Water Quality Parameters 

**Figure 1:**
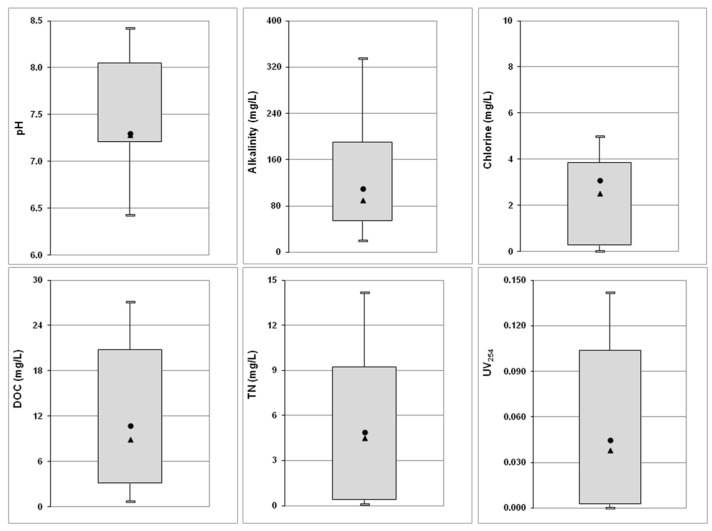
Water quality parameters in swimming pools (boxplots represent 10th and 90th percentiles, min and max values; circle and triangle represent mean and median values, respectively).

The general water quality parameters are shown in Figure 1. The pH values of waters ranged from 6.4–8.4. The majority of samples (95%) meet the local operational guideline for a range of 7.2 to 8.2 for waters treated with chlorine. Free chlorine concentrations were found to vary greatly among the investigated waters (0.3–5 mg/L). According to national legislation, free residual chlorine range is between 0.4 and 0.7 mg/L [24]. Recently, for precautionary reasons, a concentration of 1.5 mg/L is suggested for protection of public health to SARS-CoV-2 [25]. WHO suggests that a range of residual chlorine from 1 to 3 mg/L [26]. In our study most samples were within the acceptable limits proposed by WHO and only 15% of samples exceed the upper WHO guideline. The alkalinity in pool water ranged from 35–335 mg/L, and in general meet the local guideline for concentrations above 50 mg/L (only 5% were below this limit). Many studies worldwide reported deviations from national guidelines either for pH values or for free chlorine values [3].

DOC varied among pools and ranged from 0.7 to 27 mg/L. These values are within the range of values reported in other studies [1,3,27]. Total nitrogen (TN) ranged from <0.1 to 14 mg/L. Yeh et al. [28] reported an increasing accumulation of TN in swimming pool. The organic and nitrogen loads are highly variable and depend on their presence in filling water but mainly from the anthropogenic releases. Human body fluids, sweat, sebum, skin particles and hair, and personal care products contribute to both carbon and nitrogen load in pools [28,29,30,31].

### 3.2. Occurrence of Disinfection By-Products 

**Figure 2:**
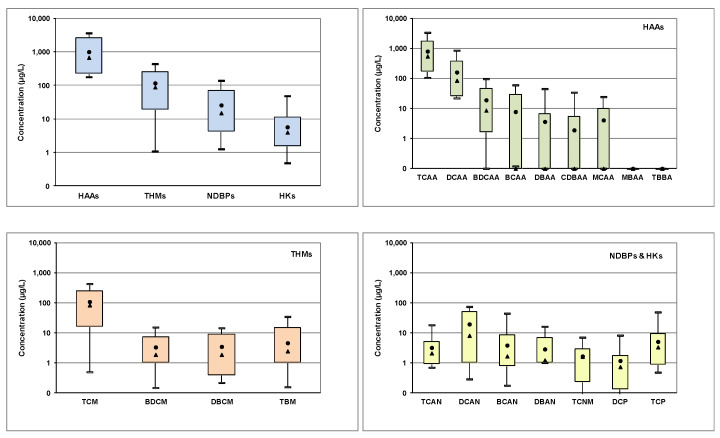
Water concentrations of DBPs in swimming pools (boxplots represent 10th and 90th percentiles, min and max values; circle and triangle represent mean and median values, respectively).

The range of various DBPs groups determined in pool water as well as of the individual species in each group is shown in Figure 2. 

HAAs exhibited the highest concentrations followed by THMs, HANs, TCNM and HKs. HAAs as sum of nine compounds ranged from 178–3640 μg/L (median 680 μg/L) (Figure 2). The dominant compounds were TCAA and DCAA with relative contributions 79 ± 15% and 18 ± 14%, respectively. BCAA, DBAA, CDBAA were determined in few samples. Yeh et al. [28] reported that due to high abundance of HAAs (especially DCAA and TCAA) these compounds could be used as indicator chemicals to define guideline values for monitoring pool water quality. The concentrations of HAAs are below ECHA proposed limits for swimming pools that ranged from 800 to 8000 μg/L for individual HAAs, and particularly 1500 μg/L for DCAA and 8000 μg/L for TCAA [32].

THMs, as sum of four compounds, was the second most abundant group with concentrations ranging from 1–410 μg/L (median 89 μg/L), with TCM being the dominant compound (84 ± 22%). Some studies compared concentrations of DBPs with drinking water limits 100 μg/L for THMs [33]. National limits have been proposed in various countries for THMs, i.e., 20 μg/L in Germany, 50 in Netherlands and Hungary [34]. ECHA proposed a limit of 50 μg/L for THMs, as chloroform equivalent, in swimming pools [32]. 

HANs as sum of four acetonitriles ranged from 0.9–130 μg/L (median 15 μg/L). DCAN (median 8.1 μg/L) and TCAN (2.1 μg/L) were the dominant compounds with relative contribution 51 + 32% and 21 + 17%, respectively, followed by BCAN, DBAN. The majority of samples exhibited concentrations below the ECHA limits for swimming pools (20 μg/L for DCAN, 70 μg/L for DBAN and 20 μg/L BCAN) [32]. TCNM ranged from <0.2–7 μg/L (median 1.6 μg/L). The haloacetone 1,1,1-TCP was the dominant propanone with concentrations 0.5–48 μg/L (median 3.4 μg/L).

The concentrations of DBPs varied among swimming pools (Figure S1) as well as within the same pool between sampling campaigns. This variation could be attributed to different conditions occurred in each swimming pool i.e., regarding low or heavy load of swimmers, replacement of pool water, filter backwashing or shock chlorination. Several outliers were found to originate mainly from two swimming pools that also exhibited high DOC values, low residual chlorine concentrations and employed electrolysis of sodium chloride as disinfection process. They also showed relatively elevated concentrations of brominated DBP species (1–12% for Br-HAAs, 13–40% for Br-THMs and 3–30% for Br-HANs). Organic load is a significant precursor of DBPs. Moreover, it has been reported that electrolysis of salt solution could result in higher formation of DBPs as well as brominated analogues due to impurities in salt [1]. 

Table 5 summarises the concentrations of DBPs in swimming pools worldwide. DBPs varied both quantitatively and in terms of speciation since their occurrence in pool water depends on operational and environmental conditions (pH, temperature, concentration and origin of organic carbon, concentration of chlorine, management conditions etc., ). The concentrations of DBPs in our study are within the reported range.

**Table 5:** Concentrations of DBPs (μg/L) in swimming pool water worldwide.

Country	HAAs	THMs	NDBPs	HKs	References
Australia	366–5126 230–2400 (DCAA) 110–2600 (TCAA)	65–84 (TCM)	4.9–8.9 (DCAN) nd–2.3 (TCNM)		[28]
Canada	155–2224				[35]
Canada		21–132 6.7–125 (TCM)	3.4–78.6 (HANs) 4.5 (TCNM)	0.3–7.3 (TCP)	[36]
China	1.2–1889	25.7 ± 33.1	12.3 ± 15.5		[37]
France		80 70 (TCM)	75 (DCAN) nd–4.5 (TCNM)	72 (TCP)	[38]
Greece	7.7–653.7	8.1–57.4	0.8–20.6 (HANs)	nd–15.3	[39]
USA	70–3980 50–2040 (DCAA) 20–2970 (TCAA)				[40]
USA		26–213 25–207 (TCM)	4–47 (DCAN)		[41]
Singapore	45–828 (DCAA) 114–1020 (TCAA)	32–170 30–167 (TCM)			[42]

nd: non detected.

Tap water from drinking water distribution system is used to fill and regularly top-up the pools at each facility. DBPs in tap water was therefore investigated. The concentrations of DBPs were low, THMs ranged from 0.4 to 17 μg/L, NDBPs <1–5 μg/L and HAAs were not found at detectable concentrations whereas DOC ranged from 0.1–1.2 mg/L. The majority of pool waters (90%) contained higher concentration of DBPs and DOC compared to filling waters. 

Correlation coefficients among the investigated DBP classes and other general water quality parameters are shown in Table S1. Significant correlations were observed among individual species in each DBP group (HAAs-DCAA-TCAA-BCAA-DBCAA-CBAA and THMs-TCM-TBM), between studied DBPs classes (THMs-HANs, HAAs-TCNM). UV was the water quality parameter that showed significant correlation with all DBPs species. Various studies reported correlation between various DBPs species and organic content or free chlorine [35,43]. However, other studies did not report significant correlations [3,40]. The origin of dissolved organic carbon in swimming pool may affect the levels and speciation of DBPs. It has been reported [27] that human body fluids exhibited higher formation potentials of HAAs than THMs, whereas the opposite was observed for the natural organic matter that produce more THMs.

### 3.3. Exposure Routes

**Figure 3:**
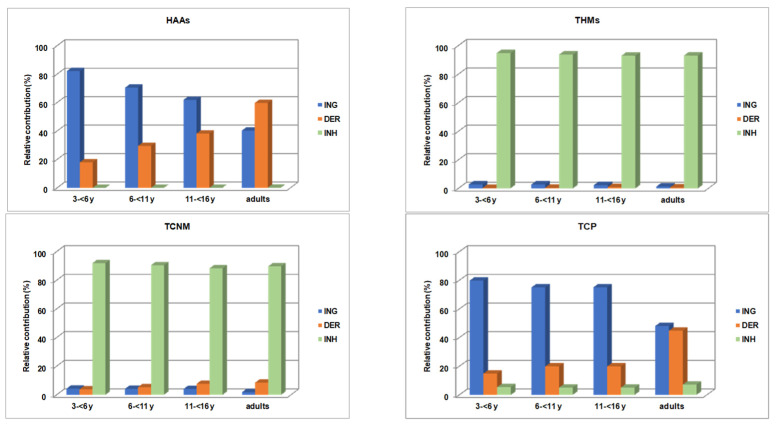
Relative contribution of exposure routes to DBPs (ING: ingestion, DER: dermal and INH: inhalation).

Ingestion, dermal contact and inhalation are the main exposure pathways to DBPs. The exposure to DBPs depends on the physical activity of swimmers and level of their effort, average time of swimming, body surface area, inhalation rate and rate of inadvertent ingestion of pool water [15,44]. The relative contribution of exposure routes to DBPs for different age-groups is shown in Figure 3. 

Route specific exposures varied among DBPs groups. Inhalation was the dominant exposure route for THMs (93–95%) and TCNM (88–92%). These compounds are highly volatile and occur in the air of natatorium. The presence of DBPs in air depends on their volatility, water concentrations, temperature of water, height above surface of swimming pool, water turbulence, humidity and air ventilation rates [16,34,45]. Inhalation was also found to be the main route of exposure to 1,1,1-TCP for children (75–80%), whereas adults exposed almost equally through ingestion and dermal absorption. Ingestion and dermal absorption were the main exposure routes for HAAs, 40–82% and 18–59%, respectively. Their relative contributing is age dependant; ingestion was the dominant exposure pathway for children, with a decreasing trend toward adults where dermal absorption became the main exposure pathway. This is because the inadvertent water intake varies with the age of swimmers, their skill and experience and type of activity. 

### 3.4. Assessment of Possible Risks 

The presence of DBPs in water and air is of major human health concern because a number of DBPs species are cytotoxic, others are carcinogenic, mutagenic or have reproductive and developmental effects [4].

#### 3.4.1. Cytotoxicity of Pool Water

**Figure 4:**
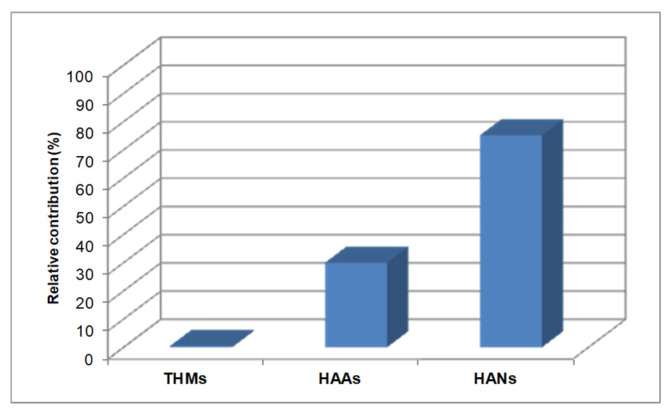
Relative contribution of DBPs groups to calculated overall cytotoxicity of water in swimming pools.

The assessment of cytotoxicity of pool water was based on the measured DBP concentrations in water and their effective concentration (EC_50_) values [13]. The relative contribution of specific DBPs groups to the total calculated cytotoxicity of pool waters is shown in Figure 4. 

THMs and HAAs contributed only to 0.5% and 24.5% of the total calculated cytotoxicity, respectively, although were the predominant DBP classes. NDBPs contributed significantly (up to 75%) to the calculated cytotoxicity, although determined at lower concentrations, Carter et al. [3] also reported low contribution of HAAs and THMs to the overall cytotoxicity of water in swimming pools whereas other DBP species such haloacetaldeydes and nitrogenous species (haloacetonitriles and haloacetamides) were the major forcing agents of toxicity. Yeh et al. [28] who actually measured cytotoxicity of pool water reported that HAAs, although the dominant DBPs, explained less than 4% of the observed cytotoxicity.

Τherefore, the occurrence of THMs and HAAs commonly measured in swimming pools cannot interpret the cytotoxicity of pool water and other DBPs species, at relatively lower concentrations, significantly contribute to this risk.

#### 3.4.2. Non-Carcinogenic and Carcinogenic Risk

Non-carcinogenic and carcinogenic risk from the exposure to THMs and HAAs was calculated since these DBPs were the most prominent compounds and their toxicity data are available (Table 4). 

**Figure 5:**
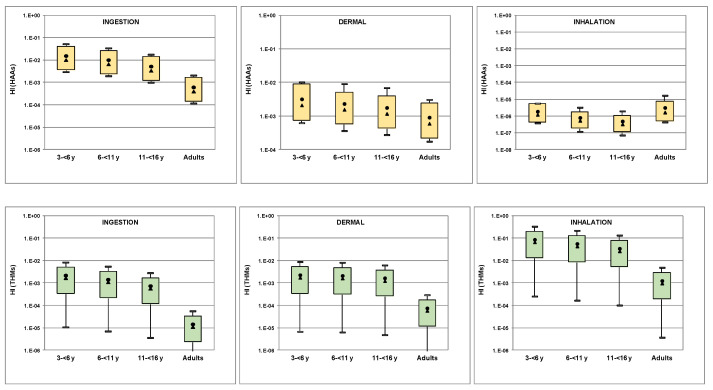
Hazard indices through different exposure routes for THMs and HAAs (boxplots represent 10th and 90th percentiles, min and max values; circle and triangle represent mean and median values, respectively).

Hazard indices for different exposure routes are illustrated in Figure 5. HI values for both THMs and HAAs from ingestion, dermal absorption and inhalation ranged from 7 × 10^−8^ to 3 × 10^−1^, well below the acceptable maximum value of 1, suggesting that non-carcinogenic risk was not of apparent concern. 

**Figure 6:**
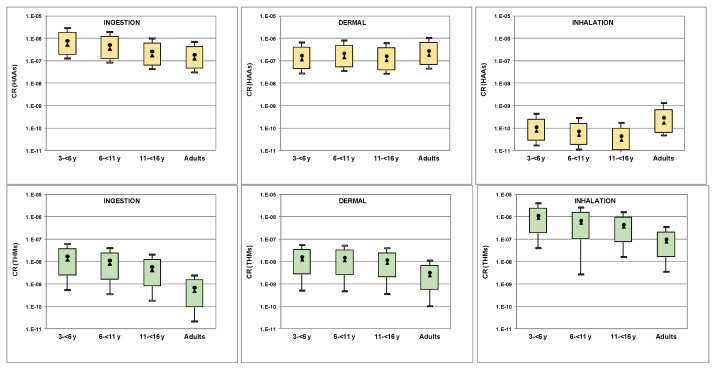
Carcinogenic risk through different exposure routes) for THMs and HAAs (boxplots represent 10th and 90th percentiles, min and max values; circle and triangle represent mean and median values, respectively).

Carcinogenic risks due to exposure to THMs and HAAs through ingestion, dermal contact and inhalation are shown in Figure 6. Inhalation posed the higher risk for THMs (4 × 10^−9^–4 × 10^−6^), with values occasionally exceeding the negligible risk limit of 10^−6^ in some facilities, whereas risk via oral and dermal routes was low. Ingestion and dermal contact posed the higher risk (3 × 10^−8^–3 × 10^−6^) for HAAs. Other studies also reported high risk posed by THMs through inhalation, often exceeding the limit of 10^−6^ [34,39,46,47]. 

There is a lot of discussion regarding calculated risks and the realistic DBPs-related risks in swimming pools. Risks could be significantly under- or over- estimated due to a number of uncertainties and assumptions that may affect the outcome. These uncertainties arise: (a) from the absence of RfDs and SFs values for each exposure route. In these cases, oral values commonly used to assess the risk via dermal exposure or inhalation. However, this extrapolation might introduce a level of uncertainty due to differences on route-specific rate and magnitude of absorption, (b) air concentrations in the natatorium calculated through different approaches, in case that are not actually measured, differ by several orders of magnitude and introduce a relevant uncertainty and, (c) other DBPs also occurred in swimming pools that may significantly contribute to realistic DBPs-related risks [34,47]. All these assumptions and uncertainties significantly affect the final risk evaluation. This urges for a common, approved methodology for risk assessment in swimming pools, for monitoring requirements of DBPs in water and indoor air quality in swimming pools facilities and for regulatory values for DBPs.

## 4. Conclusions

This study investigated the occurrence of various disinfection by-products, DBPs (THMs, HAAs, HANs, TCNM and HKs) in different types of swimming pools in the area of Thessaloniki during the period July 2019–February 2020. Moreover, water quality parameters (pH, residual chlorine, dissolved organic carbon, UV_254_ absorption, total nitrogen, alkalinity and conductivity) were measured.

The concentrations of DBPs varied among swimming pools as well as within the same pool between sampling campaigns. HAAs exhibited the highest concentrations in pool water followed by THMs, HANs, TCNM and HKs. The dominant species were TCAA, DCAA for haloacetic acids, TCM for trihalomethanes, DCAN, BCAN, DBAN and TCAN for haloacetoniriles. The majority of pool waters contained higher concentrations of DBPs and dissolved organic carbon and total nitrogen compared to filling waters. Exposure doses by ingestion, dermal absorption and inhalation for four age groups (3–<6 y, 6–<11 y, 11–<16 y and adults) were calculated. Inhalation was the dominant exposure route for THMs (93–95%) and TCNM (88–92%). Ingestion and dermal absorption were the main exposure routes for HAAs, 40–82% and 18–59%, respectively. Their relative contribution was age dependant; ingestion was the dominant exposure pathway for children, with a decreasing trend towards adults where dermal absorption became the main exposure pathway. 

HANs, although present at lower concentrations, significantly contributed, up to 75%, to calculated cytotoxicity of pool waters. The estimation of DBPs-related health risk was based on the measured water concentrations and the respective calculated air concentrations. Hazard indices for different exposure routes were very low suggesting non-carcinogenic risk. Inhalation posed the higher carcinogenic risk for THMs, with values occasionally exceeding the negligible risk limit of 10^−6^, whereas risk via oral and dermal routes was low. Ingestion and dermal contact posed the higher risk for HAAs although at lower levels. Due to uncertainties and assumptions in the risk assessment process, further studies are needed to comprehensively evaluate the extent and the acceptability of risks to DBPs in swimming pools. Risk management strategies that minimise the exposure to DBPs without compromising disinfection efficiency as well as the development of health-based guidelines are necessary.

## Figures and Tables

**Table 1:** DBPs groups determined in this study.

DBPs Groups	DBPs Species	Abbreviation	Chemical Formula
THMs Trihalomethanes	trichloromethane	TCM	CHCl_3_
	dichlorobromomethane	DCBM	CHBrCl_2_
	chlorodibromo	CDBM	CHBr_2_Cl
	methanetribromomethane	TBM	CHBr_3_
HANs Haloacetonitriles	dichloroacetonitrile	DCAN	CHCl_2_CN
	trichloroacetonitile	TCAN	CCl_3_CN
	bromochloroacetonitrile	BCAN	CHBrClCN
	dibromoacetontrile	DBAN	CHBr_2_CN
HNMs Halonitromethanes	trichloronitromethane	TCNM	CCl_3_NO_2_
HKs	1,1-dichloropropanone	DCP	C_3_H_4_Cl_2_O
Haloketones	1,1,1-trichloropropanone	TCP	C_3_H_3_Cl_3_O
HAAs Haloacetic acids	monochloroacetic acid	MCAA	CH_2_ClCOOH
	monobromoacetic acid	MBAA	CH_2_BrCOOH
	dichloroacetic acid	DCAA	CHCl_2_COOH
	trichloroacetic acid	TCAA	CCl_3_COOH
	bromochloroacetic acid	BCAA	CHBrClCOOH
	dibromoacetic acid	DBAA	CHBr_2_COOH
	dibromochloroacetic acid	DBCAA	CBr_2_ClCOOH
	chlorodibromo acetic acid	CDBAA	CBr_2_ClCOOH
	tribromoacetic acid	TBAA	CBr_3_COOH

**Table 2:** Description of swimming pools.

Code	Indoor/Outdoor	Children/Adults	Disinfection
SP-1	Indoor	Children ^1^	NaOCl
SP-2	Indoor	Children/Adults ^2^	NaOCl
SP-3	Outdoor	Children/Adults	NaOCl
SP-4	Outdoor	Children/Adults	NaOCl
SP-5	Indoor	Children	NaOCl
SP-6	Indoor	Children/Adults	NaOCl
SP-7	Outdoor	Children/Adults	NaOCl
SP-8	Outdoor	Children/Adults	NaOCl
SP-9	Outdoor	Children/Adults	NaOCl
SP-10	Indoor	Children	Electrolysis NaCl
SP-11	Indoor	Children/Adults	Electrolysis NaCl
SP-12	Indoor	Children/Adults	Electrolysis NaCl
SP-13	Outdoor	Children	NaOCl
SP-14	Outdoor	Children/Adults	NaOCl

^1^ swimming pools only for children ≤7 years old, ^2^ Swimming pools for children >7 and adults.

## Supplementary Materials

The following are available online. Figure S1: Concentrations of DBPs (mean ± sd) in swimming pools. Table S1: Spearman’s correlation coefficients (values in bold were significant at 0.01 level, values in italics were significant at 0.05 level).

## References

[1] Carter R.A.A., Joll C.A. (2017). Occurrence and formation of disinfection by-products in the swimming pool environment: A critical review. J. Environ. Sci..

[2] Lyas H., Masih I., van der Hoek J.V. (2018). Disinfection Methods for Swimming Pool Water: Byproduct Formation and Control. Water.

[3] Carter R.A.A., Allard S., Croué J.P., Joll C.A. (2019). Occurrence of disinfection by-products in swimming pools and the estimated resulting cytotoxicity. Sci. Total Environ..

[4] Richardson S.D., Plewa M.J., Wagner E.D., Schoeny R., DeMarine D.M. (2007). Occurrence, genotoxicity, and carcinogenicity of regulated and emerging disinfection by-products in drinking water: A review and roadmap for research. Mutat. Res..

[5] Richardon S.D., Postigo C. (2016). Discovery of New Emerging DBPs by High-Resolution Mass Spectrometry. Compreh. Anal. Chem..

[6] Stalter D., O’Malley E., von Gunten U., Escher B.J. (2016). Fingerprinting the reactive toxicity pathways of 50 drinking water disinfection by-products. Water Res..

[7] Villanueva C.M., Cordier S., Font-Ribera L., Salas L.A., Levallois P. (2015). Overview of Disinfection By-products and Associated Health Effects. Curr. Environ. Health. Rpt..

[8] Font-Ribera L., Marco E., Grimalte J.O., Pastor S., Marcos R., Abramsson-Zetterberg L., Pedersen M., Grummt T., Junek R., Barreiro E. (2019). Exposure to disinfection by-products in swimming pools and biomarkers of genotoxicity and respiratory damage–The PISCINA2 Study. Environ. Int..

[9] Couto M., Bernard A., Delgado L., Drobnic F., Kurowski M., Moreira A., Rodrigues-Alves R., Rukhadze M., Seys S., Wiszniewska M. (2021). Health effects of exposure to chlorination by-products in swimming pools. Allergy.

[10] U.S. EPA (1995). Method 551.1 Determination of Chlorination Disinfection Byproducts, Chlorinated Solvents, and Halogenated Pesticides/Herbicides In Drinking Water By Liquid-Liquid Extraction And Gas Chromatography with Electron-Capture Detection.

[11] U.S. EPA (2003). Method 552.3 Determination of Haloacetic Acids and Dalapon In Drinking Water by Liquid-Liquid Microextraction, Derivatization and Gas Chromatography with Electron Capture Detection.

[12] APHA (2012). Standard Methods for the Examination of Water and Waste Water.

[13] Wagner E.D., Plewa M.J. (2017). CHO cell cytotoxicity and genotoxicity analyses of disinfectionby-products: An updated review. J. Environ. Sci..

[14] U.S. EPA (2003). Swimmer Exposure Assessment Model (SWIMODEL).

[15] U.S. EPA Exposure Assessment Tools by Routes. https://www.epa.gov/expobox/exposure-assessment-tools-routes.

[16] Dyck R., Sadiq R., Rodriguez M.J., Simard S., Tardif R. (2011). Trihalomethane exposures in indoor swimming pools: A level III fugacity model. Water Res..

[17] Lourencetti C., Grimalt J.O., Marco E., Fernandez P., Font-Ribera L., Villanueva C.M., Kogevinas M. (2012). Trihalomethanes in chlorine and bromine disinfected swimming pools: Air-water distributions and human exposure. Environ. Int..

[18] U.S. EPA (2011). Exposure Factors Handbook.

[19] R.A.I.S Risk Assessment Information System (RAIS) Online Database. https://rais.ornl.gov/cgi-bin/tools/TOX_search?select=chemspef.

[20] PubChem. National Center for Biotechnology Information. U.S. National Library of Medicine, USA. https://pubchem.ncbi.nlm.nih.gov/compounds.

[21] U.S. EPA (2014). The USEPA Integrated Risk Information System (IRIS) Online Database. Washington, DC, USA. http://www.epa.gov/iris/subst/index.html.

[22] I.A.R.C. International agency for Research of Cancer. Agents Classified by the IARC Monographs, Volumes 1–128. https://monographs.iarc.fr/list-of-classifications.

[23] O.E.H.H.A. California Office of Environmental Health Hazard Assessment. https://oehha.ca.gov/chemicals/chloroform.

[24] (1973). Greek National Legislation Γ1/443/1973 On Swimming Pools Following Instructions for Their Construction and Operation.

[25] (2020). Greek National Legislation F.Ε.Κ. 21.5.2020, (Δ1(δ)/ΓΠοικ 32179/2020) Regarding Measures for Protection of Public Health In The Context Of Avoiding Spread Of SARS-Cov-2 After Commissioning Of Swimming Pools.

[26] WHO (2006). Guidelines for Safe Recreational Water Environments.

[27] Kanan A., Karanfil T. (2011). Formation of Disinfection By-Products in Indoor Swimming Pool Water: The Contribution from Filling Water Natural Organic Matter and Swimmer Body Fluids. Water Res..

[28] Yeh R.Y., Farre M.J., Stalter D., Tang J.Y., Molendijk J., Escher B.I. (2014). Bioanalytical and chemical evaluation of disinfection by-products in swimming pool water. Water Res..

[29] Keuten M.G.A., Peters M.C.F.M., Daanen H.A.M., de Kreuk M.K., Rietveld L.C., van Dijk J.C. (2014). Quantification of continual anthropogenic pollutants released in swimming pools. Water Res..

[30] Tsamba L., Cimetière N., Wolbert D., Correc O., Le Cloirec P. (2020). Body fluid analog chlorination: Application to the determination of disinfection byproduct formation kinetics in swimming pool water. J. Environ. Sci..

[31] Wang J., Gong T., Xian J. (2020). Formation of haloacetic acids from different organic precursors in swimming pool water during chlorination. Chemosphere.

[32] European Chemical s Agency (2017). Guidance on The BPR: Vol V Disinfection By-Products. ECHA-17-G-01-EN..

[33] European Parliament (2020). EU Directive 2020/2184 Of the European Parliament and of The Council of 16 December 2020 On the Quality Of Water Intended For Human Consumption. OJ. L 435/1, 23.12.2020.

[34] Pándics T., Hofer A., Dura G., Vargha M., Szigeti T., Tóth E. (2018). Health risk of swimming pool disinfection by-products: A regulatory perspective. J. Water Health.

[35] Simard S., Tardif R., Rodriguez M.J. (2013). Variability of chlorination by-product occurrence in water of indoor and outdoor swimming pools. Water Res..

[36] Tardif R., Catto C., Haddad S., Simard S., Rodriguez M. (2016). Assessment of air and water contamination by disinfection by-products at 41 indoor swimming pools. Environ. Res..

[37] Peng F., Peng J., Li H., Li Y., Wang B., Yang Z. (2020). Health risks and predictive modeling of disinfection byproducts in swimming pools. Environ. Int..

[38] Manasfi T., De Méo M., Coulomb B., Di Giorgio C., Boudenne J.L. (2016). Identification of disinfection by-products in freshwater and seawater swimming pools and evaluation of genotoxicity. Environ. Int..

[39] Kargaki S., Iakovides M., Stephanou E. (2020). Study of the occurrence and multi-pathway health risk assessment of regulated and unregulated disinfection by-products in drinking and swimming pool waters of Mediterranean cities. Sci. Total Environ..

[40] Wang X., Garcia L., Zhang H., Yang H., Yuefeng X. (2014). Haloacetic acids in swimming pool and spa water in the United States and China. Front Environ. Sci. Eng..

[41] Kanan A., Selbes M., Karanfil T. (2015). Occurrence and formation of disinfection by-products in indoor US swimming pools. ACS Symp. Ser..

[42] Yang L., Schmalz C., Zhou J., Zwiener C., Chang V.W., Ge L., Pun M. (2016). An insight of disinfection by-product (DBP) formation by alternative disinfectants for swimming pool disinfection under tropical conditions. Water Res..

[43] Zhang X., Yang H., Wang X., Zhao Y., Wang X., Xie Y. (2015). Concentration levels of disinfection by-products in 14 swimming pools of China. Environ. Sci. Eng..

[44] Font-Ribera L., Kogevinas M., Schmalz C., Zwiener C., Marco E., Grimaltf J.O., Liu J., Zhang X., Mitch W., Critelli R. (2016). Environmental and personal determinants of the uptake of disinfection by-products during swimming. Environ. Res..

[45] Nitter T.B., Svendsen K.H. (2019). Modelling the concentration of chloroform in the air of a Norwegian swimming pool facility—A repeated measures study. Sci. Total Environ..

[46] Chowdhury S. (2015). Predicting human exposure and risk from chlorinated indoor swimming pool: A case study. Environ. Monit. Assess..

[47] Gouveia P., Felgueiras F., Mourão Z., Fernandes E.D.O., Moreira A., Gabriel M.F. (2019). Predicting health risk from exposure to trihalomehtanes in an Olympic-size indoor swimming pool among elite swimmers and coaches. J. Toxicol. Environ. Health A.

