# Supplementary material for: Occurrence of Disinfection By-Products in Swimming Pools in the Area of Thessaloniki, Northern Greece. Assessment of Multi-Pathway Exposure and Risk
Source: Molecules. 2021 Dec 16;26(24):7639. doi: 10.3390/molecules26247639 (PMC8703401; doi:10.3390/molecules26247639)
Supplement: Supplementary file 1 [file molecules-26-07639-s001.zip › molecules-1478658-supplementary.pdf]

SUPPLEMENTARY MATERIAL

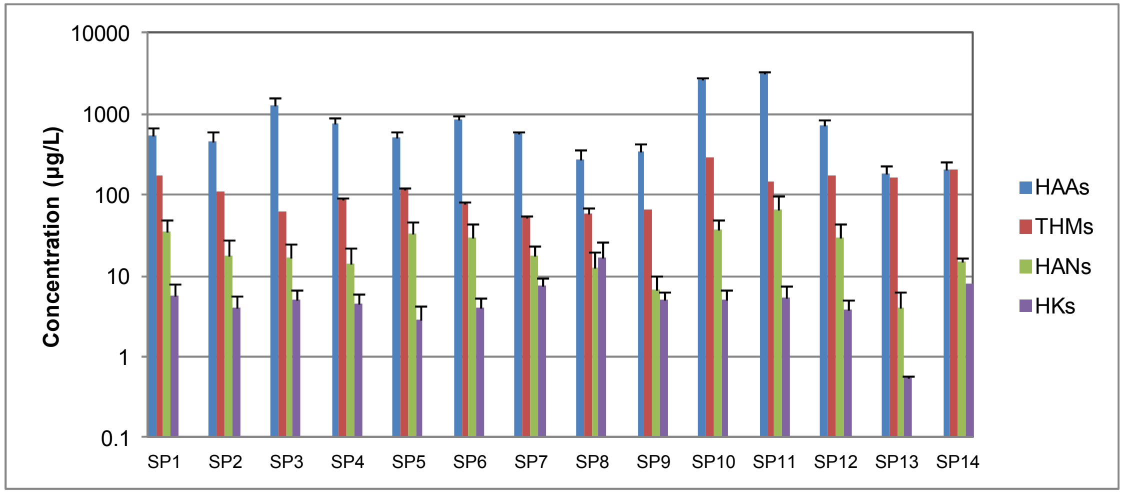

Figure S1. Concentrations of DBPs (mean±sd) in swimming pools**.**

Table S1. Spearman's correlation coefficients (values in bold are significant at 0.01level, values in italics are significant at 0.05 level)

	HAAs	DCAA	TCAA	BCAA	DBAA	BDCAA	CDBAA	THMs	TCM	BDCM	DBCM	TBM	HANs	TCAN	DCAN	BCAN	DBAN	TCNM	TCP	Cl2	DOC	UV	SUVA	pH	Alk	TN
HAAs	**1**																									
DCAA	**0.887**	**1**																								
TCAA	**0.992**	**0.845**	**1**																							
BCAA	**0.412**	**0.451**	**0.385**	**1**																						
DBAA	**0.551**	**0.558**	**0.531**	**0.626**	**1**																					
BDCAA	**0.922**	**0.835**	**0.92**	**0.437**	**0.575**	**1**																				
CDBAA	**0.539**	**0.534**	**0.521**	**0.491**	**0.818**	**0.539**	**1**																			
THMs	-0.158	-0.114	-0.187	*0.325*	*0.324*	-0.105	**0.392**	**1**																		
TCM	-0.170	-0.127	-0.199	*0.334*	*0.310*	-0.115	**0.357**	**0.993**	**1**																	
BDCM	**0.774**	**0.813**	**0.748**	**0.411**	**0.590**	**0.749**	**0.558**	0.003	-0.033	**1**																
DBCM	0.194	0.232	0.156	*0.311*	**0.516**	0.205	**0.473**	**0.444**	**0.435**	**0.401**	**1**															
TBM	-0.222	-0.217	-0.216	0.118	0.124	-0.240	0.255	**0.375**	*0.309*	-0.092	-0.047	**1**														
HANs	0.103	0.130	0.068	**0.407**	0.245	0.217	0.178	**0.517**	**0.524**	0.214	**0.401**	0.019	**1**													
TCAN	-0.056	-0.018	-0.064	0.124	*0.265*	-0.144	**0.346**	**0.382**	*0.331*	0.097	0.107	**0.711**	0.082	**1**												
DCAN	0.180	0.207	0.149	**0.389**	0.195	*0.282*	0.157	**0.447**	**0.466**	*0.264*	**0.437**	-0.136	**0.880**	-0.158	**1**											
BCAN	**0.453**	**0.528**	**0.422**	**0.402**	**0.430**	**0.541**	**0.340**	0.156	0.149	**0.594**	**0.517**	-0.198	**0.618**	-0.125	**0.629**	**1**										
DBAN	**0.475**	**0.503**	**0.446**	0.226	**0.524**	**0.405**	**0.471**	-0.022	-0.047	**0.656**	**0.489**	-0.028	0.108	0.188	0.103	**0.484**	**1**									
TCNM	**0.410**	**0.409**	**0.414**	0.246	**0.409**	*0.332*	**0.502**	0.153	0.122	0.602	0.243	0.084	0.006	0.199	0.106	0.221	**0.390**	**1**								
TCP	0.075	0.018	0.073	0.243	-0.047	0.197	-0.042	0.112	0.159	**0.072**	0.076	*-0.27*	**0.526**	*-0.324*	**0.608**	*0.331*	-0.030	-0.112	**1**							
Cl2	-0.042	-0.288	0.012	-0.281	**-0.484**	-0.216	**-0.484**	-0.185	-0.153	*-0.338*	*-0.354*	-0.376	0.022	-0.077	0.031	-0.270	-0.226	-0.177	0.247	**1**						
DOC	0.234	0.202	0.210	0.183	*0.312*	0.159	*0.278*	0.117	0.109	0.230	0.092	0.152	-0.031	0.254	-0.175	-0.085	*0.298*	0.068	-0.113	0.086	**1**					
UV	**0.473**	**0.755**	*0.368*	**0.479**	**0.709**	**0.526**	**0.683**	**0.703**	**0.663**	**0.590**	**0.547**	**0.434**	**0.493**	**0.591**	**0.476**	**0.467**	*0.418*	**0.431**	-0.177	**-0.472**	0.275	**1**				
SUVA	0.305	**0.499**	0.260	0.278	*0.403*	*0.355*	**0.424**	**0.511**	**0.462**	*0.381*	0.267	**0.447**	0.322	**0.476**	*0.351*	0.302	0.078	*0.349*	-0.225	*-0.404*	-0.193	**0.823**	**1**			
pH	0.111	0.067	0.094	-0.157	0.069	0.018	0.061	-0.020	-0.038	0.177	0.148	-0.132	-0.169	-0.113	-0.081	-0.067	0.178	**0.406**	*-0.267*	0.055	0.255	-0.037	-0.201	**1**		
Alk	0.147	0.217	0.109	0.012	0.186	0.056	0.156	0.201	0.155	*0.278*	0.138	0.257	-0.072	**0.348**	-0.175	0.005	0.199	0.227	**-0.599**	-0.119	**0.450**	*0.419*	0.217	**0.429**	**1**	
TN	**0.493**	**0.400**	**0.519**	0.186	0.111	0.461	0.146	**-0.419**	**-0.447**	*0.319*	-0.230	-0.078	**-0.363**	-0.054	**-0.377**	0.071	0.162	0.213	*-0.259*	0.041	0.165	-0.225	-0.008	0.104	0.115	**1**
